# A novel method for classifying cortical state to identify the accompanying changes in cerebral hemodynamics

**DOI:** 10.1016/j.jneumeth.2016.04.005

**Published:** 2016-07-15

**Authors:** R. Slack, L. Boorman, P. Patel, S. Harris, M. Bruyns-Haylett, A. Kennerley, M. Jones, J. Berwick

**Affiliations:** a Department of Psychology, University of Sheffield, Western Bank, Sheffield S10 2TN, United Kingdom; b Department of Systems Engineering, University of Reading, Whiteknights, Reading RG6 6AY, United Kingdom

**Keywords:** Neurovascular coupling, Brain state classification, Hemodynamics

## Abstract

**Background:**

Many brain imaging techniques interpret the haemodynamic response as an indirect indicator of underlying neural activity. However, a challenge when interpreting this blood based signal is how changes in brain state may affect both baseline and stimulus evoked haemodynamics.

**New method:**

We developed an Automatic Brain State Classifier (ABSC), validated on data from anaesthetised rodents. It uses vectorised information obtained from the windowed spectral frequency power of the Local Field Potential. Current state is then classified by comparing this vectorised information against that calculated from state specific training datasets.

**Results:**

The ABSC identified two user defined brain states (synchronised and desynchronised), with high accuracy (∼90%). Baseline haemodynamics were found to be significantly different in the two identified states. During state defined periods of elevated baseline haemodynamics we found significant decreases in evoked haemodynamic responses to somatosensory stimuli.

**Comparison to existing methods:**

State classification – The ABSC (∼90%) demonstrated greater accuracy than clustering (∼66%) or ‘power threshold’ (∼64%) methods of comparison.

Haemodynamic averaging – Our novel approach of selectively averaging stimulus evoked haemodynamic trials by brain state yields higher quality data than creating a single average from all trials.

**Conclusions:**

The ABSC can account for some of the commonly observed trial-to-trial variability in haemodynamic responses which arises from changes in cortical state. This variability might otherwise be incorrectly attributed to alternative interpretations. A greater understanding of the effects of cortical state on haemodynamic changes could be used to inform techniques such as general linear modelling (GLM), commonly used in fMRI.

## Highlights

• We classified brain state using a vector-based categorisation of neural frequencies.
• Changes in cerebral blood volume (CBV) were observed when brain state altered.
• During these state alterations, changes in blood oxygenation were also found.
• State dependent haemodynamic changes could affect blood based brain imaging.

## 1 Introduction

It is unclear whether, and to what extent, cerebral haemodynamics are modulated by spontaneous changes in cortical state. This is important for the accurate interpretation of perfusion-related imaging signals, such as Blood Oxygen Level Dependent Functional Magnetic Resonance Imaging (BOLD fMRI). BOLD fMRI infers the location and magnitude of neural responses to stimuli or cognitive tasks by exploiting a process known as neurovascular coupling, in which concurrent alterations in the local demand for glucose and oxygen during neuronal activation are accompanied by regional changes in cerebral blood flow (CBF), blood volume (CBV) and oxygenation (Attwell et al., 2010, Kleinfeld et al., 2011, Masamoto and Kanno, 2012). Within such techniques, brain responses to identical stimuli routinely exhibit considerable trial to trial variability in their timing and magnitude (Duann et al., 2002). This information may well be behaviourally meaningful, but a full understanding of its origins is currently lacking.

Since haemodynamic responses to stimuli/task presentations are often small, typically representing a slight fractional change from baseline conditions, a common approach in brain imaging studies is to average across multiple stimulus evoked trials in order to enhance the signal to noise ratio (SNR) (Boorman et al., 2010, Martin et al., 2013). However, such methods implicitly assume that the baseline brain state remains constant during the experiment, which may not be appropriate. Indeed, changes in brain state have been widely reported in both human and animal studies and have been linked to alterations in sleep cycles (Steriade et al., 2001, Castro-Alamancos, 2004b, Gervasoni et al., 2004, Timofeev et al., 2012), affect (Nakic et al., 2006), alertness/arousal (Junghöfer et al., 2006, Poulet and Petersen, 2008, Stoelzel et al., 2009, Poulet et al., 2012) and attention (Kastner et al., 1999, O'Connor et al., 2002, Kohn et al., 2009, Rossi and Pourtois, 2012). Changes in state have also been shown to alter the processing of sensory stimuli (Castro-Alamancos, 2004b), modulate the delicate equilibrium between cortical excitation and inhibition (Taub et al., 2013) and to be correlated with variability of encoding into working memory (Myers et al., 2014) therefore making identification of brain state a prerequisite for accurate interpretation of how sensory stimuli are represented in the brain. Furthermore, both baseline and stimulus evoked haemodynamics have been shown by our laboratory to be altered during changes in cortical state actively induced by direct stimulation of the brainstem (Jones et al., 2008), intravenous infusion of psychostimulants (Berwick et al., 2005), and during hypercapnia challenge (Kennerley et al., 2012). Notwithstanding these insights, the question remains as to whether haemodynamics are altered during spontaneous changes in brain state.

The somatosensory cortex of the anaesthetised rodent allows invasive concurrent optical measures of cortical haemodynamics and electrophysiological recordings to be made. Furthermore, urethane anaesthesia is an ideal model with which to address this, as it induces cyclic and spontaneous changes in cortical state, as observed in the ongoing Local Field Potential (LFP) (Friedberg et al., 1999). Indeed, since the 1950s (Moruzzi and Magoun, 1949) it has been known that the cortex can interchange between states of quiescence and activation (Castro-Alamancos, 2004a). These two states were traditionally referred to as synchronised (large amplitude low frequency neuronal oscillations) and desynchronised (high frequency low amplitude neuronal oscillations) (Steriade et al., 1993, Lampl et al., 1999, Steriade et al., 2001). Although many different brain states can be experienced, Harris and Thiele (2011) suggest brain states change over a continuum from the synchronised to the desynchronised state. Therefore for simplicity, we have chosen to initialise the Automatic Brain State Classifier (ABSC) on only the synchronised and desynchronised states. Classification of states purely by observation presents certain challenges. For example, if the subject does not change brain state for the length of an experiment, it can be hard for the observer to classify based on a single LFP amplitude, as absolute values can vary from animal to animal. State classification can also be a lengthy process and may elicit boredom or tiredness in the observer, and thus errors could occur. The need to classify brain states has been acknowledged, and some methods for separation are already in existence that show evidence for identifying different states, and state transitions (Gervasoni et al., 2004, Michel et al., 2012). However, as these methods are dependent upon techniques such as supervised clustering of the data, they typically require large quantities of data in order to provide a robust and reliable result. The use of a neural marker to classify states reduces the need for large amounts of data, as state can be identified whenever the marker is found. We have made a formal comparison between our novel technique which uses a set of neural markers, a clustering technique, and a ‘power threshold’ of the broadband LFP signal.

Here, we present the Automatic Brain State Classifier (ABSC), a novel method that efficiently identifies neural markers to classify different brain states. The ABSC was initialised using spontaneous LFP data recorded from the whisker barrel region of the somatosensory cortex in the urethane anaesthetised rat. Once initialised, the ABSC is unsupervised, and is a stable, absolute classifier able to detect state from small time periods of data without needing observation of a state change before classification. During the desynchronised state, the ABSC is able to detect an increase in the power of the upper spectral frequencies relative to the lower spectral frequencies. These identified shifts in spectral mass were associated with a significant increase in the baseline haemodynamics and a decrease in the stimulus evoked haemodynamic response.

Our results confirm and extend previous work by our laboratory and others. We demonstrate the ability of the ABSC to conduct post-experiment state classification (with the potential to conduct online state classification), and highlight the importance of accounting for spontaneous variations in brain state during neuroimaging studies in the anaesthetised rodent.

## 2 Method

In this paper, we describe a novel method to automatically classify neural brain states in order to evaluate the coupled haemodynamics. The method for data collection is described, followed by an explanation of the novel analysis technique and finally the comparison to two existing standard methods is described. The ABSC is initialised on LFP data where synchronised and desynchronised states are both present.

### 2.1 Data collection

#### 2.1.1 Animal preparation and surgery

All experiments were performed in accordance with the Animal (Scientific Procedures) Act 1986, with approval from the United Kingdom Home Office. Neural and haemodynamic responses were collected from adult female Hooded Lister rats weighing (200–350 g). Rats were kept in a 12 h light/dark cycle and allowed access to food and water ad libitum*.* After being briefly anaesthetised with isoflurane, the animals were intraperitoneally injected with 1.25 g/kg urethane. After the injection, a homoeothermic heating blanket (Harvard Instruments, UK) with rectal monitoring was subsequently used to maintain the core body temperature at 37° C until the termination of the experiment. Atropine was administered at 0.4 mg/kg subcutaneously to decrease mucous secretions during surgery. To allow artificial ventilation (Harvard Instruments, UK) and to monitor end-tidal CO_2_ recordings (CapStar-100, CWE Systems, USA), the animals were tracheotomised. The cannulation of the femoral artery and vein permitted the monitoring of the mean arterial blood pressure (MABP) and the infusion of phenylephrine (0.13–0.26 mg/h) respectively, ensuring MABP was kept between 100 and 110 mmHg (Golanov et al., 1994, Nakai and Maeda, 1999). The arterial cannulation also allowed blood samples to be taken for measurement of blood oxygen saturation. This ensured that the ventilator parameters could be adjusted to maintain the animal within normal physiological limits. Animals were placed in a Kopf Instruments stereotaxic frame, and the surface of the head was exposed. Following this, the right side of the skull was thinned to translucency using a dental drill. Optical translucency was maintained by affixing a plastic well with dental cement to the edge of window and infusing through the well a continuous supply of saline.

#### 2.1.2 Concurrent electrophysiology and 2D optical imaging spectroscopy

2D-OIS was used to measure the haemoglobin concentration and blood oxygen saturation across the surface of the somatosensory (S1) cortex. The imaging procedure followed that detailed in (Berwick et al., 2008) and therefore will only be briefly described here. A CCD camera (Dalsa 1M30P, USA) recorded images at 32 Hz from the cortical surface. Four wavelengths (495 ± 31, 559 ± 16, 575 ± 14, and 587 ± 9 nm, full-width half-maximum) with contrasting absorption coefficients were used by a high-speed Lambda DG-4 filter changer, to illuminate the cortex, giving an effective image sampling rate of 8 Hz. From this multi-wavelength data, estimates of total haemoglobin (Hbt), oxyhaemoglobin (Hbo) and deoxyhaemoglobin (Hbr) were obtained using a modified Beer–Lambert law (Berwick et al., 2005).

An initial optical imaging spectroscopy experiment was conducted to localise the whisker barrel region. Haemodynamic responses were evoked in the whisker barrel cortex, through electrical stimulation of the whisker pad, further detail is described in the application protocol below. The haemodynamic data was analysed using a standard GLM approach, to localise the region overlying the whisker barrel somatosensory cortex, activated by the stimuli.

The resultant ‘activation map’ was used to guide the insertion of a 16 channel linear array electrode (NeuroNexus technologies, USA) perpendicular to the whisker barrel cortical surface. The electrode was inserted into, and normal to, the cortex to a depth of 1500 mm (i.e. approximately layer VI) and sampling occurred at 24.414 kHz. This data was then downsampled to 1.53 kHz. The electrode had 16 channels in total with 100 mm spacing, site area 177 mm^2^, 1.5–2.7 MW impedance, and 33 mm tip width (Neuronexus Technologies, Ann Arbor, MI, USA). Concurrent electrophysiological and 2D-OIS (haemodynamic) data were recorded. To examine the 2D-OIS data over time, a region of interest (ROI) was selected, centered on the electrode, and extended to capture the cortical region that was associated with the stimulus evoked haemodynamics, elicited in the initial experiment. The average ROI size for an animal was 515 pixels with a SD of 202.6 (1 pixel = ∼49 mm^2^).

#### 2.1.3 Overview of application protocols

Three datasets were used; the ABSC was initialised using the first dataset and comparisons between the ABSC and exisiting state sorting methods were made. The second and third datasets were then used to examine both the effectiveness of the ABSC and to examine the stability of the ABSC over long periods of time. The three datasets are described in more detail below.

##### 2.1.3.1 (A1, *n* = 12) spontaneous recordings

Concurrent neural and haemodynamic changes were recorded concurrently for 1000–2500 s, in the absence of stimuli, from 12 subjects. A subset of the recordings were used to initialise the ABSC, before the ABSC was applied to classify the whole dataset. The ABSC classification of A1 was then compared to the ‘expert’ classification of A1 (see Section 2.2.2) to check classification timings and accuracy.

##### 2.1.3.2 (A2, *n* = 14) electrical stimulation of the whisker pad

The initialised ABSC was applied to concurrent neural and haemodynamic recordings, acquired during electrical stimulation of the whisker pad. The classifications arising from the ABSC were again compared to an ‘expert’ classification. Stimulation of the whisker pad was evoked through two insulated stainless steel electrodes (2 mm exposed tip) inserted subcutaneously into the whisker pad between rows A/B and C/D. Electrical stimulation (0.8–1.2 mA; 300 μs pulse width, 16 s duration at 5 Hz) caused a visible full pad whisker twitch confirming that stimulation was effective. This stimulation caused no changes to the MABP or CO_2_ recordings. Haemodynamic and neural signals were recorded concurrently for 2100 s, split into 30 trials of 70 s inter-trial interval with the stimulus applied after 10 s.

##### 2.1.3.3 (A3, *n* = 5) electrical stimulation of the brainstem reticular formation

The third dataset used neural recordings presented previously (Jones et al., 2008), where brainstem stimulation was applied to actively change brain state. Details of the exact procedure can be found in Jones et al. (2008). In brief, the method of data collection followed that detailed above although recordings of neural and haemodynamics were not concurrent. Modulation of cortical state was produced in 4 out of 5 animals by direct electrical stimulation of the brainstem reticular formation (2 s duration, 200 Hz, stimulation current: <200 μA, pulse duration: 3 ms). In this paper, we examine only neural recordings for this dataset. We present Fig. 7 for the reader to visually judge ABSC performance.

### 2.2 Data analysis

The ABSC method to classify the different brain states consists of three main components; Feature Extraction, Model Vector Definition and Model Vector Comparison. The main components are described in detail below.

#### 2.2.1 Feature Extraction

A Fast Fourier Transform (FFT) was applied to raw neural recordings from channels 13 to 16 (∼1200–1500 μm, below the cortical surface, approximately layer Vb and VI according to depth information found in Wright and Fox (2010) or Meyer et al. (2013)), which were split with a moving window of length 10 s (overlapped by 1/10 of the window size—1 s step), giving a frequency resolution of 0.1 Hz. This was used to extract spectral information from the neural recordings. A different window size and overlap was used for initialisation and is detailed in Section 2.2.2 below. The spectral power was then subdivided into the five main frequency bands: Delta (*δ*, 0.5–3 Hz), Theta (*θ*, 4–7 Hz), Alpha (*α*, 8–12 Hz), Beta (*β*, 13–30 Hz) and Gamma (*γ*, 31–80 Hz), to obtain frequency power time series for each band (*f*_i_) and each channel (13–16). The information from channels 13 to 16 of the electrode was then averaged together. Electrode channels 13–16 were used for all ABSC analysis, throughout this paper, as the deeper channels have previously been shown to give the best signal power, especially in some of the EEG bands such as delta (Rappelsberger et al., 1982, Sirota et al., 2003).

#### 2.2.2 Model Vector Definition

All animals and experiments in the ‘spontaneous’ dataset (A1) were classified by a human ‘expert’ observer. To classify, the ‘expert’ examined the entire time series visually (in a MATLAB figure window, with the ability to adjust the zoom function) and the points in time at which state changes occurred, based on reductions in the broadband LFP amplitude, were recorded using a custom written MATLAB (The Mathworks, USA) script. The ‘expert’ observer also indicated if the state was stable for the entire experiment, and if so, in what state the experiment remained. We defined an ‘expert’ here as someone who has previously published work on brain states.

**Fig. 2:**
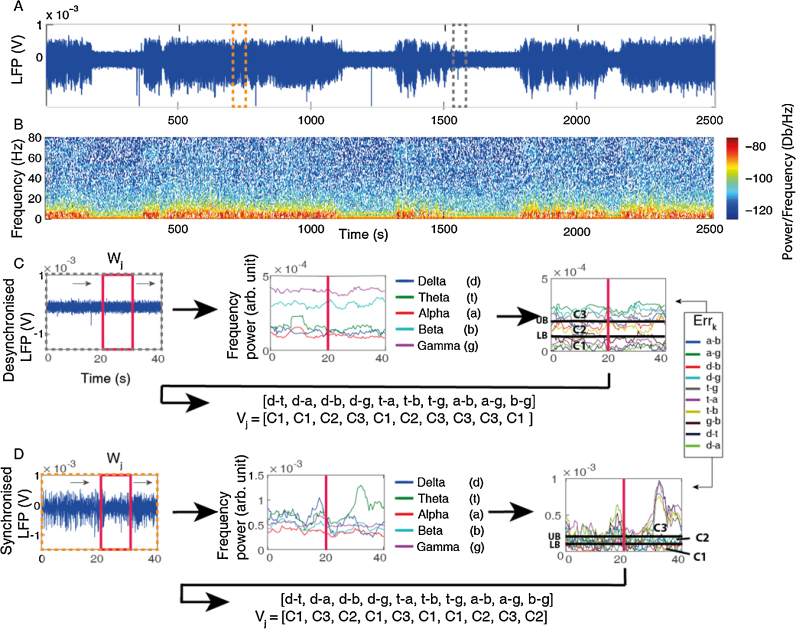
(A) Exemplar raw training data showing LFP recording (channel 13–16, averaged) with clear distinction between the two states within the data. (B) Fourier power spectrogram of the LFP data in A. (C) Obtaining coded vectors from desynchronised training data. A sliding window Fourier transform was applied to the neural recording to calculate the classical EEG bands (middle). The red vertical lines mark the time point for the frequency power band information obtained from the moving window (left, red box). The EEG bands were then subtracted from one another to give relative frequency information. This information was coded with regards to an upper bound (UB) and a lower bound (LB), see Model Vector Definition, point 2 for how the bounds were obtained and point 3 for coding. An example of the coded error vector that would be obtained at the time point of the red line is then given below. (D) As C, but with synchronised training data. Note, UB and LB are the same as C, this is necessary to keep coding of the vectors consistent. (For interpretation of the references to colour in this figure legend, the reader is referred to the web version of this article.)

Approximately 10% of this dataset was designated as a training dataset (a single full experiment, Fig. 2A) and was used to generate the model vectors (M*_l_*) for the state classifier. The training dataset was split into synchronised and desynchronised states defined by the ‘expert’ observer and the feature extraction step performed. The only change to the feature extraction step detailed above (Section 2.2.1) is that a 4 s window with a 0.4 s step was used in initialisation (giving a frequency resolution of 0.25 Hz). We used a smaller window during initialisation to capture more of the time variations present in the raw data as at this stage, more detail with regards to time variation was more valuable than processing speed. After initialisation the larger window (10 s, 1 s step) was used as the reduced processing time was deemed to be more valuable than the finer time resolution, especially when processing larger datasets. Each state subset of the training dataset was examined as follows, with the desynchronised subset examined first:

(1) A simple measure of the relation of each frequency band power to the other four bands was calculated using subtraction of each frequency power time series from the other bands frequency power time series, ensuring each band was only examined in relation to the others once (i.e. combinations were used, so alpha is subtracted from delta but delta is not subtracted from alpha).

(1) $$\begin{array}{c} f_{i}=\left[\delta ,\theta ,\alpha ,\beta ,\gamma \right]\\ {\text{Err}}_{k}=f_{i}-f_{i+1}\;\;\forall 1\leq i\leq 5,k=1:10 \end{array}$$

This produces a set of ten time series (Err*_k_*), providing a measure of the relation of each frequency band power to the others, rather than an absolute frequency power measure. Each point (*j*) in each time series represents a measure of a pair of relative frequencies for a particular window of LFP data. The absolute value of each data point is then taken to negate the importance of the order of subtraction.

(2) $$\begin{array}{cc} {\text{Err}}_{kj}=|({\text{Err}}_{kj})| & j\geq 1 \end{array}$$

(2) Upper (UB) and lower (LB) error bands were automatically set as follows:

(3) $$\begin{array}{c} UB=mean\left(\left|\text{mean}\left({\text{Err}}_{k}\right)\right|)(\text{Rounded to the nearest decimal place}\right)\\ LB=0.5\times UB \end{array}$$

These bounds can only be set once, as consistency in the coded vectors needs to be maintained. They should be set from the state subset of training data that shows the smallest variance in frequency power. In this paper, the bounds were set in the desynchronised state as this had smaller variance in frequency power than the synchronised state (Fig. 2D).

(3) Each data point in each of the subtracted time series was then coded (CErr*_kj_*) to classify it, based on its relation to the upper and lower bounds. This gives three possible codes for each point.

(4) $$\begin{array}{ccc} \text{Case 1:} & \text{If}{\;\;\;\;\text{Err}}_{kj}<\text{LB} & {\text{CErr}}_{kj}=\text{C}1\\ \text{Case 2:} & \text{If}\;\;\;\text{LB}{\leq \text{Err}}_{kj}\leq \text{UB} & {\text{CErr}}_{kj}=\text{C2}\\ \text{Case 3:} & \text{If}{\text{Err}}_{kj}>\text{UB} & {\text{CErr}}_{kj}=\text{C3} \end{array}$$

At any time point (*j*), this therefore gives a 10 point coded vector (V*_j_*) representing the 10 subtracted frequency band pairs for a window of LFP data (see Fig. 2C for example). Here, we set C1 = 2, C2 = 3 and C3 = 4, but any integer separated coding could be used to the same effect.

(4) The *l* most frequently occurring coded vectors in the desynchronised classified state were set as the model vectors (M*_l_*), where *l* vectors explained 76.4% of the coded vector variance in the desynchronised state. The same number of model vectors were selected for the synchronised state to maintain a consistent comparison, although this number explained less vector variance (8.4%). These vectors were defined as the model vectors.

#### 2.2.3 Model Vector Comparison

The data (∼90%) not used to train the ABSC was used to test the state classifier. Steps 1 and 3 from the Model Vector Definition were performed. Each coded vector (V*_j_*) was then compared to the model vectors (M*_l_*) from each state with the best fit selected as the state, desynchronised or synchronised, for that window of LFP data (W*_j_*).

(5) $$\begin{array}{cc} {\text{SW}}_{j}=\underset{\text{l}}{\text{min}}\left(\Sigma \left|V_{j}-M_{l}\right|\right) & {\text{SW}}_{j}\in \left\{\text{desynchronised, synchronised}\right\} \end{array}$$

#### 2.2.4 Details of the application of the ABSC to the experimental datasets

(A1) The A1 Dataset (spontaneous) was used in initialising the ABSC and testing whether there were differences in the concurrently recorded haemodynamic changes, between the two brain states inferred from the LFP. This dataset was also used for the comparison with the two existing methods of classification.

The model vectors from the initial analysis (A1) were kept constant during A2 and A3 to check the stability of the vectors.

(A2) The A2 test dataset (‘stimulus evoked’) was subject to the above feature extraction and pattern classification steps with a minor amendment. During the feature extraction step, time points during the 16 s period of stimulation (−0.001–16.432 s for each trial) were ignored to prevent the interference of the stimulus in the spectral analysis. Once the ABSC had been applied to each subject dataset, trials were classified using only a pre-stimulation period of varying length (1–10 s). We varied the length of the pre-stim period to observe how the accuracy of the ABSC changed as it was given decreasing amounts of data with which to classify. The most common state was found for this pre-stim time period and the trial was classified as belonging to this state. Accuracy levels of the ABSC compared to the ‘expert’ classified trials were calculated.

(A3) The A3 dataset (‘electrical stimulation of the brainstem reticular formation’) followed the feature extraction and pattern classification steps. Again, the period of stimulation was not subject to classification by the ABSC.

#### 2.2.5 Sectioning of concurrent haemodynamic data

We describe how the ABSC was used to partition the concurrent haemodynamic data for each of the applicable experimental datasets.

(A1) If the time period of a cortical state detected from the neural recordings was of long duration (>30 s), then the concurrently measured haemodynamics were examined over the same time period. These long duration haemodynamic time periods were then averaged across time to obtain the mean level of Hbo, Hbr and Hbt for each state.

(A2) The individual 70 s stimulation trials were averaged according to the state classification from the neural activity, during the pre-stimulus baseline period and again the concurrent haemodynamics (Hbo, Hbr and Hbt) were examined for each state. An inclusion criteria was specified, to reduce potential noise from subjects that did not have many simulation trials in a specific state. Thus for an experimental run to be included in the overall state classified haemodynamic averaged, it must be found to provide at least 5 trials in that particular state.

### 2.3 Method comparison

The ABSC was compared to two other typical state sorting methods, one based on a popular clustering methodology taken from the paper by Gervasoni et al. (2004), the other using a simple ‘power threshold’ of the raw LFP signal similar to that used by Papanicolaou et al. (1986). The A1 ‘spontaneous’ dataset was used for these methods.

#### 2.3.1 Comparison to a clustering technique

We followed the methodology shown in the Gervasoni et al. (2004) paper to cluster the A1 dataset. We are grateful to the authors for kindly supplying their code for this comparison, which we used for the more complicated analysis after extracting the spectral ratio and performing the principal component analysis (PCA). The only alteration we made to their methodology was to decrease the window size, as our dataset was much smaller than the dataset they used. We used a 1 s window with a 0.5 s step, rather than the 2 s window with a 1 s step that was used in their original paper.

#### 2.3.2 Comparison to a ‘power threshold’ technique

A simple ‘power threshold’ technique was applied to the raw LFP data within the A1 dataset, and as the threshold required for this technique is based on absolute power it was recalculated for each experiment.

*Obtaining the* ‘*power threshold*’

1. LFP Data were obtained from channels 13–16 within a moving window of size 10 s (stepped by 1 s) to extract raw power information from these neural recordings.
2. To obtain the RMS of each temporal window, we squared each data point within the window, took the mean of these points, and then took the square root of the mean.
3. Data were averaged across channels 13–16.
4. The threshold for each individual experiment was calculated. We took the average value of all the RMS windowed data points for the experiment and set this number as the individual state threshold for the experiment.

RMS windowed data points that were higher than the individual threshold were classified as synchronised and points that were lower than this threshold were classified as desynchronised.

## 3 Results

The ABSC was used to classify temporal periods of neural recordings into two brain states. These classifications were then used to investigate whether a change in brain state caused changes in baseline and stimulus evoked haemodynamics. The accuracy of the ABSC was compared to two other methods of state sorting.

### 3.1 The initialisation of the ABSC using spontaneous data (dataset A1) and comparison to alternative techniques

**Fig. 3:**
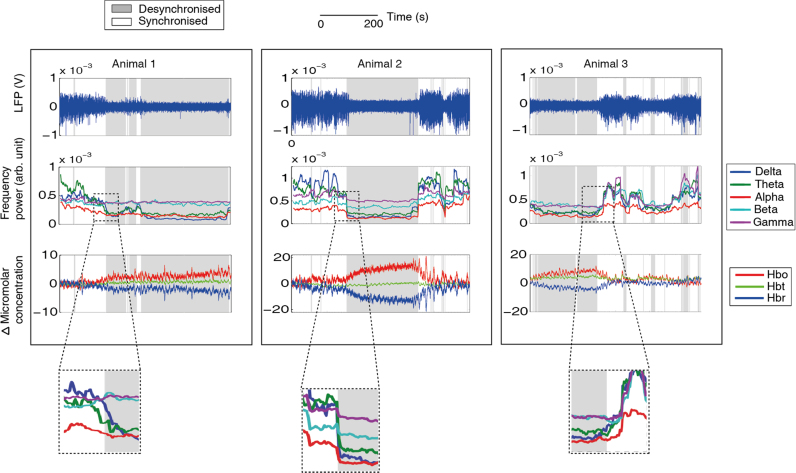
The application of the ABSC demonstrating classification of concurrently recorded neurovascular coupled spontaneous data. 500 s of data shown for each example animal. Top line: LFP with classified section shaded in grey for desynchronised brain state and white for synchronised (repeated throughout figure). Middle: demonstrates feature extraction step of the method, the windowed frequency bands are plotted continuously on the same time scale as the neural and haemodynamic data. Bottom: concurrent haemodynamic time series showing micromolar changes in Hbo, Hbr and Hbt from baseline levels. Insets indicate a zoomed view of the ratio changes in the frequency bands as the cortical state changes. (For interpretation of the references to colour in this figure legend, the reader is referred to the web version of this article.)

Data were collected from 13 experiments across 12 animals, where no stimulus was applied. Each experiment involved the concurrent recording of neural and haemodynamic data for durations between 1100–2500 s. Each animal contributed one experiment to the experimental dataset. One animal also provided an additional experiment which was used solely for training data. The ABSC was trained on state separated data previously classified by an ‘expert’. The five most frequently occurring error vectors (M*_l_*), resulting from initialising the ABSC, explained 76.4% of the coded vector variance in the desynchronised state spectral information from the training dataset and were set as the desynchronised model vectors. To keep the number of vectors consistent, the five most frequently occurring vectors in the synchronised state were also set as the model vectors for the state sorter, giving a total of 10 model vectors. Once the model vectors were selected, the ABSC was applied to the experimental dataset. A demonstration of the application of the ABSC for state sorting is shown for three subjects in Fig. 3.

Visual inspection of the classification of the ABSC of the spontaneous neural activity, demonstrates its ability to capture the periods of cortical state change (Fig. 3, top row). The frequency band spectral information (Fig. 3, middle row) reflects an increase in the ratio of the upper relative to the lower spectral frequencies for the desynchronised state, whereas in the synchronised state, this ratio decreased or disappeared. A clear difference in the relational frequencies can be observed (Inset boxes below Fig. 3), with gamma and beta high relative to alpha, delta and theta in the desynchronised state, whilst all frequencies except alpha were in close proximity in the synchronised state.

We have systematically evaluated the accuracy of the ABSC across all 12 animals by comparing the ABSC classified state time periods to ‘expert’ classified state time periods on a point by point basis (Table 1, row two). We have also compared the performance of the ABSC to the performance of two alternative methods of classification, a clustering technique (Table 1, row three) and a ‘power threshold’ technique (Table 1, row four). The alternative methods were assessed in the same way as the ABSC by comparison to ‘expert’ classified state time periods on a point by point basis.

The Clustering method had a slightly higher accuracy than the ABSC on the data points it assigned to a particular cluster (Table 1, column 2), however, when the amount of data it was unable to classify was taken into account, the total accuracy was greatly reduced compared to that of the ABSC (Table 1, column 4). The clustering method was often unable to identify periods of desynchronised data, only succeeding when large amounts of very clearly distinct data were present in the desynchronised state. In contrast, as the ABSC classifies on a point by point basis, it did not have this drawback. The ‘power threshold’ technique also had a greatly reduced accuracy rate when compared to the ABSC (Table 1, column 4), again this technique suffered when the neural data did not have even temporal periods in both of the two states. Thus, the ABSC presents clear advantages over the other two techniques and so we continue our analysis of the concurrently recorded haemodynamic data using the ABSC as the state classifier.

**Fig. 4:**
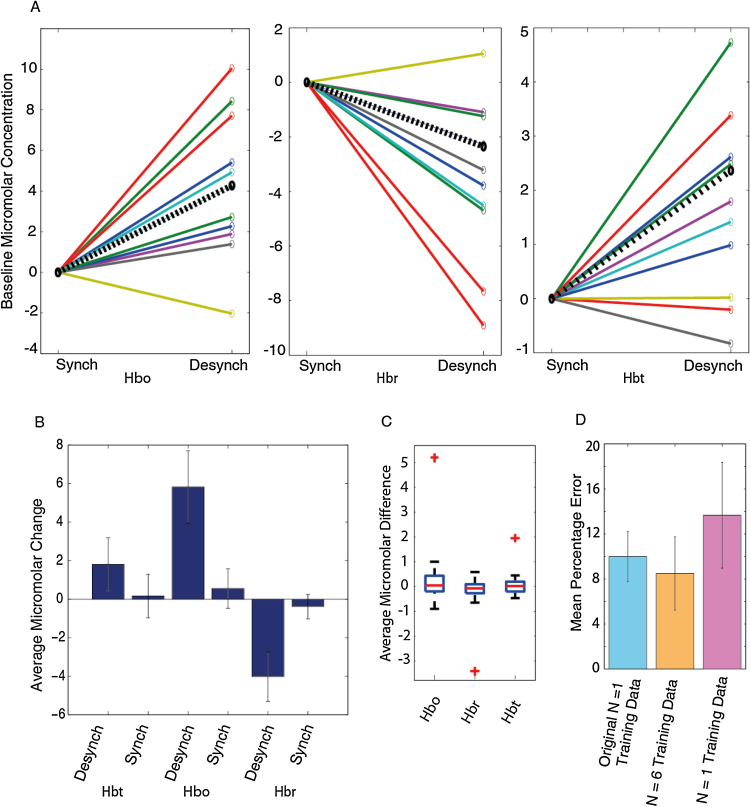
Baseline haemodynamic changes observed during periods of classified state in the absence of stimuli (A1). (A) Changes in Hbo (left), Hbr (middle) and Hbt (right) between synchronised (S) and desynchronised (D) states for all animals. Synchronised state values have been normalised to 0 to allow observation of the changes in baseline concentrations. Dotted black lines show the change averaged across all animals. (B) Average micromolar changes in Hbt, Hbo and Hbr across all animals with error bars showing standard error of the mean. (C) Boxplot denoting variation in spontaneous haemodynamic averages from randomised periods of synchronised and desynchronised time periods (control analysis, randomisation classifications were performed 50 times and ABSC classifications were included in plotted data). For each state, the average across animals was found and then the desynchronised average was subtracted from the synchronised. On each box, the central red line is the median, the edges of the box are the 25th and 75th percentiles, the whiskers extend to the most extreme data points not considered outliers, and outliers (defined by the MATLAB boxplot function) are plotted individually (red crosses—these mark the ABSC classified averages). (D) Variation in the accuracy of the ABSC by using alternative training datasets to initialise the model vectors (original animal, *N* = 6 average training set, alternative single animal training dataset). (For interpretation of the references to colour in this figure legend, the reader is referred to the web version of this article.)

The ABSC classified time periods of synchronised and desynchronised state were used to extract the concurrent haemodynamics (Fig. 3, bottom row). Clear increases in Hbo and Hbt can be seen, with a drop in Hbr during extended time periods of desynchronisation. During these periods, the levels of Hbt rose to a peak and remained elevated. Decreases back to baseline were identified by the ABSC as occurring during the synchronised time periods. Three animals were classified as having no state changes, and so were excluded from further analysis. Across the remaining 9 animals, we examined the levels of Hbo, Hbr and Hbt (Fig. 4A). Only time periods where the neural activity was classified as being in a single state stable for longer than 30 s were included, since shorter time periods would render data more vulnerable to noise distortions and the effects of state transitions. The average baseline change of Hbt was significantly larger during desynchronised, rather than synchronised states (Fig. 4B, *p* = 0.0104, t-test, *p* corrected for multiple comparisons). Hbo was also significantly larger (*p* = 0.0025, *t*-test *p* corrected) and Hbr was significantly lower (*p* = 0.01, *t*-test *p* corrected) during these respective states.

In order to exclude the possibility that the training data chosen could have unduly influenced the results of the ABSC, we initialised the ABSC using both training data from an alternative individual animal (within A1) and additionally, training data from an average of 6 animals (the experimental dataset being the 7 remaining experiments from the other 6 animals in A1) and repeated our assessment of state sorting accuracy. The mean percentage error when comparing the ABSC results to the ‘expert’ sort using alternative training datasets were similar, with only one animal providing an outlying accuracy measure for both alternative datasets (Fig. 4D). A repeated measures ANOVA revealed no significant variation in accuracy between the training datasets (*F*(1.131, 12.44) = 0.694, *p* = 0.438, Greenhouse–Geisser values used as Mauchley’s test of sphericity was significant), indicating that the accuracy of the ABSC was independent of the training datasets used.

To ensure the haemodynamic effects found were not different by chance, we randomly assigned the time periods identified by the ABSC to either the synchronised or desynchronised state for each animal, then averaged within and across animals for the two states, again using only periods of 30 s+ stable state. This was then repeated 50 times and the results compared to the ABSC averages (Fig. 4C).The results indicate that the differences in the haemodynamic baseline between the two cortical states are not due to chance as the ABSC difference was the only outlier in each box plot analysis. Thus, we have demonstrated that the state sorter can effectively classify LFP data into two states and this classification has a significant effect on the levels of concurrently measured Hbo, Hbr and Hbt during spontaneous recordings.

### 3.2 Classification of cortical state and the implications for associated haemodynamics using a stimulus evoked dataset 16 s 5 Hz (A2)

Using the same ABSC initialisation settings as (A1), we used the ABSC to classify the state of neural activity, recorded during 16 s 5 Hz electrical stimulation of the whisker pad. This allows for the examination of the effect of cortical state on the stimulus-evoked neurovascular responses. Neural and haemodynamic data were recorded during 30 stimulus presentation trials, with 70 s between each stimulus presentation. In addition to the classification of the entire time series of neural recordings (as in A1), we also applied the ABSC to 10 s, 5 s and 1 s pre-stimulation baseline neural information to predict the cortical state of each trial.

The state classifier took 1085 s to classify all 16 s 5 Hz stimulation data (14 animals and 24 experiments, giving a total of 875 min of data) on our lab computer (4 core, 4.2 GHz). Again comparisons were made on a single window basis between ‘expert’ classified data and the ABSC, this gave the classifier an accuracy of 88.6% (SD = 8.54%). On this occasion, the ‘expert’ was unable to correctly classify 3 experiments from 3 animals where the state was invariant. Based on the ratio of the upper and lower spectral frequencies, it appeared the ABSC made the correct classification of these experiments (see S1, Fig. A.1 for further information in Supplementary material) and examination of the time-course of haemodynamic changes also reinforced the ABSC classification. The state invariant experiments were therefore removed from the overall accuracy measure for the 16 s stimulation data (*n* = 13) and highlight the importance of using an automatic classifier.

**Fig. 5:**
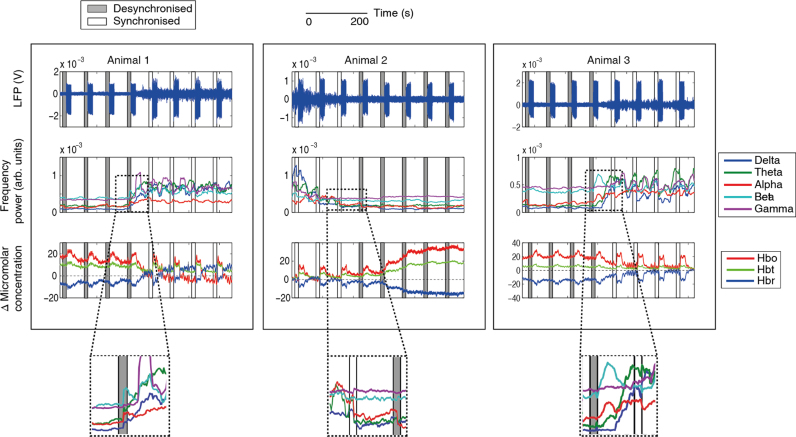
The predictive classification of stimulus evoked trials by the ABSC for concurrent neural and haemodynamic data to 3 exemplar animals which have been subject to 16 s electrical stimulation of the whisker pad. Eight individual stimulation trials of 70 s length are shown for each example animal. Top line: LFP with pre-stimulus period, used by the ABSC, boxed and shaded in grey for desynchronised brain state and white for synchronised (repeated throughout figure). Middle line: demonstrating the feature extraction step of the method, the windowed frequency bands are plotted continuously on the same time scale as the neural and haemodynamic data. Bottom line: concurrent haemodynamic time series showing micromolar changes in Hbo, Hbr and Hbt from baseline levels. Insets indicate a close view of the changes in the frequency band power as the cortical state alters. (For interpretation of the references to colour in this figure legend, the reader is referred to the web version of this article.)

Variable amplitude oscillations can be observed, during the non-stimulation periods, in the time course of the neural responses (see Fig. 5, top row, for exemplar subjects demonstrating clear periods of synchronised and desynchronised state in their neural recordings). Periods of desynchronisation are marked by decreases in the amplitude of LFP oscillations (Fig. 5, trials marked with gray sections), when compared to synchronised periods (Fig. 5, trials marked with white sections). Spectral frequency power analysis of the same desynchronised periods shows the characteristic increases in the ratio of higher to lower spectral frequencies (Fig. 5 middle row), as seen in the exemplar spontaneous data (Fig. 3). During the same periods of desynchronisation, increases in the baseline Hbt and Hbo and decreases in Hbr can also be observed (Fig. 5, bottom row), when compared to synchronised periods. In contrast to the baseline haemodynamic changes, the stimulus evoked haemodynamic changes show an inverse relationship, whereby responses evoked during periods classified as desynchronised show evoked Hbo, Hbr and Hbt responses that are attenuated, or greatly reduced, whilst responses evoked during periods classified as synchronised appear robust, with clear differences from baseline present in Hbo, Hbr and Hbt. The large differences in evoked neural and haemodynamic responses across the different states could have a large effect on the accurate interpretation of neuroimaging data, we have therefore compared averaging across all stimulation trials to averaging selectively by using the cortical state sorter.

#### 3.2.1 Comparison with standard averaging of evoked trials, without accounting for state

**Fig. 6:**
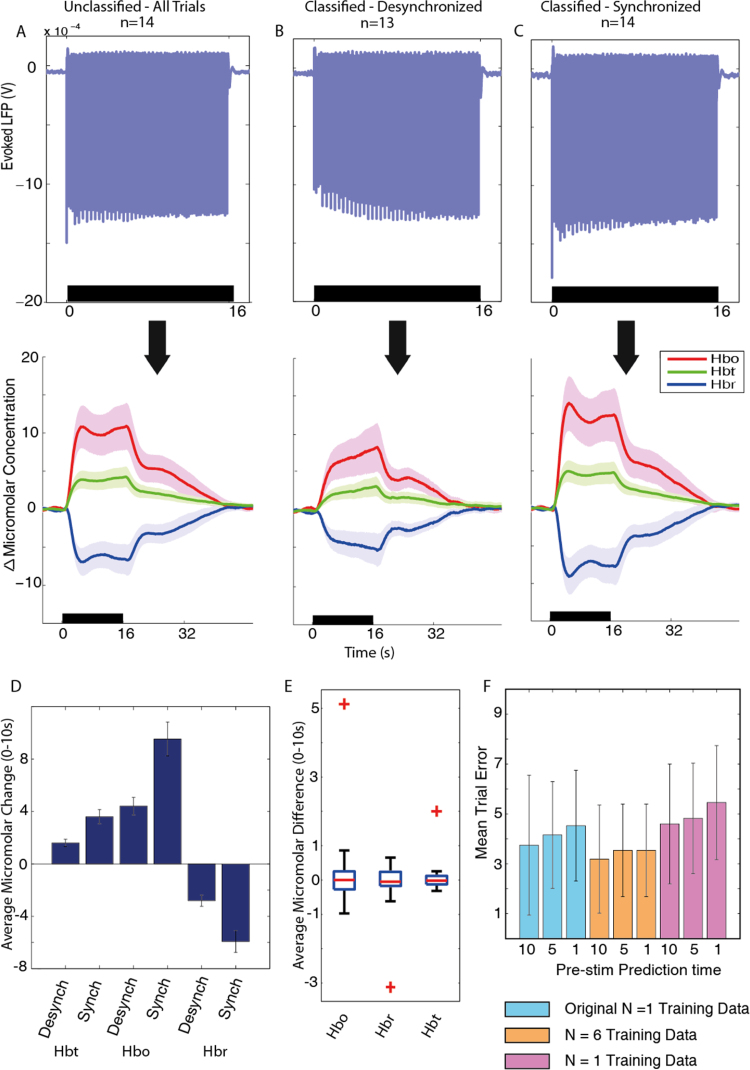
Application of the ABSC to neurovascular coupled 16 s 5 Hz whisker pad stimulation data. (A) Undivided averaged responses from all trials, from all animals (neural activity top left and haemodynamics bottom left). The error patches on the haemodynamic figures show 1 unit of standard deviation for each time series. (B) Average responses from all stimulation trials classified in the desynchronised brain state (*n* = 13, one animal did not show a desynchronised time-period) average neural activity (top) and haemodynamic responses (middle). (C) Average responses from all stimulation trials classified in the synchronised brain state (*n* = 14) average neural activity (top) and haemodynamic responses (middle). (D) State classified micromolar changes in Hbt, Hbo and Hbr, averaged over 0–10 s after stimulation and averaged across all subjects, with error bars showing 1 unit of standard error. (E) Box plot denoting variation in evoked haemodynamic averages from 50 randomised trial selection datasets. For each randomised trial set, the average across animals was found and then the desynchronised average was subtracted from the synchronised. For each box, the central red line is the median, the edges of the box are the 25th and 75th percentiles, the whiskers extend to the most extreme data points not considered to be outliers, and outliers (defined by the MATLAB box plot function) are plotted individually (red crosses mark the ABSC classified trial set). (F) Variation in the accuracy of the predictive trial onset by period of time provided to the ABSC (10, 5 and 1 s) as well as by initialisation of ABSC by alternative training dataset (original animal, *N* = 6 average training set and alternative single animal training dataset). Horizontal black bars denote stimulation period. (For interpretation of the references to colour in this figure legend, the reader is referred to the web version of this article.)

To better understand how the state classification of neural data and the subsequent classification of the concurrent haemodynamics affects evoked responses, we examined average neurovascular responses across all animals for trials classified as desynchronised (Fig. 6B) and synchronised (Fig. 6C) and compared our state classified averages with the standard method of averaging across all trials for all animals (Fig. 6A). Following on from our investigation of the effects of cortical state on baseline haemodynamics, we calculated that the average change in baseline haemodynamics for the desynchronised state was an increase of 8.56 μM for Hbo, 2.00 μM for Hbt and a decrease of 5.56 μM for Hbr, when compared to the synchronised state. These changes altered the initial parameter assumptions for the desynchronised state, giving a new micromolar concentration of 106 and blood saturation of 58% (Fig. 6B and C reflect these parameters). The average neural responses (Fig. 6, top row) show distinct differences after the onset of stimulation, for the different conditions. Stimulus evoked LFP depolarisations in the desynchronised state (Fig. 6, top row, second column), start with a small magnitude, but then increase gradually in magnitude with the subsequent stimulation pulses. Stability in the magnitude of the LFP depolarisations is achieved approximately 10–12 s after stimulus onset. Conversely in the synchronised state, the initial LFP depolarisation is of greater magnitude (Fig. 6, top row, third column), followed by a fast return to a smaller more stable LFP magnitude, approximately 8–10 s after stimulus onset. The averaged unclassified neural response can be seen to be a combination of the neural responses from desynchronised and synchronised classified trials. In contrast to the desynchronised LFP responses, the standard average response shows a maximal LFP on the first response, although the magnitude of this is not as large as the synchronised initial LFP. The subsequent LFPs reach a steady magnitude by approximately 9–10 s after stimulation onset.

The stimulus evoked haemodynamic changes accompanying the changes in neural activity have magnitudes which are modulated in a similar manner to that observed for the state sorted neural activity. Haemodynamic responses in the synchronised state (Fig. 6C, bottom) display a larger initial peak around 5 s after stimulus onset, followed by a subsequent plateau and return to baseline, whilst evoked haemodynamic changes during desynchronised periods (Fig. 6B bottom) do not show an initial peak and reach their maximum around 16 s after stimulus onset, before returning to baseline.

Evoked Hbt responses were significantly larger during the synchronised state compared to the desynchronised state (Fig. 6D, *p* = 0.00023, *t*-test, corrected for multiple comparisons), Hbo was also significantly larger (*p* = 0.00018, *t*-test, corrected) and Hbr was significantly lower (*p* = 0.00011, *t*-test, corrected) during these respective states.

To ensure the haemodynamic effects found were not significantly different due to chance, we randomly assigned haemodynamic trials to either the synchronised or desynchronised state for each animal, and then averaged within and across animals for the two states (Fig. 6E). This was then repeated 50 times and the results were plotted in a box plot including the real ABSC averages. We used the standard formula to determine outliers in this dataset of 51 averages: x>Q3+1.5×IQRorx<Q1+1.5×IQR, where *x* is the average micromolar difference, IQR is the inter-quartile range and *Q*1 and *Q*3 are the 25th and 75th percentiles respectively. The ABSC averages were the only outliers in this data group, indicating that the effect of extracting and averaging sections of haemodynamic data is reliant upon our method of classification.

We examined the effect on classification accuracy of varying the time period of data the ABSC was applied to, before each stimulus onset, to predict the classification of the state. We found that a decrease in the amount of time allowed for predictive trial classification, did cause a slight drop in the accuracy of the sorter, but this was not significantly different (Fig. 6F, *p* = 0.399, one way ANOVA). We also found that using an alternative single animal or average of 6 animals to create the initialisation training dataset (the animals used for Fig. 4D) had virtually no effect on the overall classification accuracy rates. To further validate the accuracy and to assess the ability of the ABSC to sort non-spontaneously state shifting data, we applied the ABSC to a previously published dataset, which artificially changed cortical state through direct stimulation of the brain stem and is described below.

### 3.3 Electrical stimulation of the brainstem reticular formation—application dataset (A3)

**Fig. 7:**
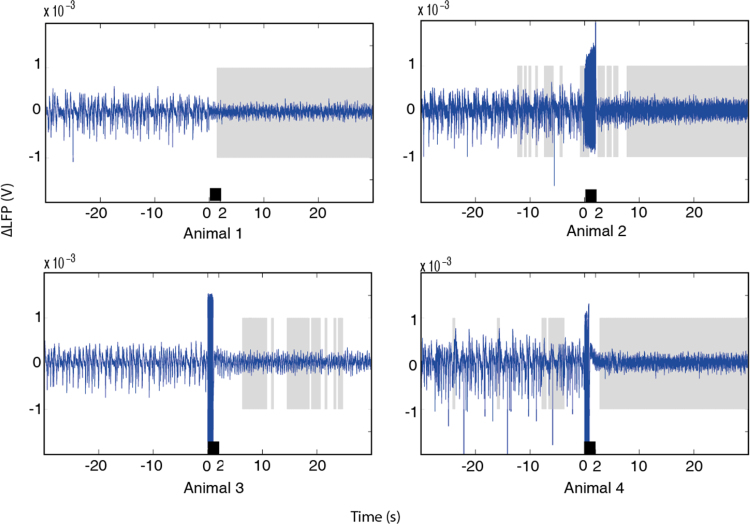
The application of the ABSC to 4 animals from the dataset of Jones et al. (2008). Neural activity with ABSC classified sections shaded in grey for desynchronised brain state and white for synchronised. The black bars denote the duration of time that the reticular stimulation was applied. Stimulation artefacts can be seen in 3 out of 4 animals. (For interpretation of the references to colour in this figure legend, the reader is referred to the web version of this article.)

The ABSC was also tested on data from Jones et al. (2008) to check the stability of the model vectors. Jones et al., stimulated the brainstem directly to produce a cortical desynchronisation, so the time at which the state change took place was artificially fixed and allowed for a further validity check. Using the same initialisation settings as in (A1), the ABSC successfully identified the brainstem stimulation in 4 out of 5 cases (Fig. 7), demonstrating clear stability. In the 5th case, the ABSC did not find a clear change in state, however upon evaluation by eye, it appeared that the cortical state had not been artificially changed in this case (see S2 Fig. A.2 for further information in Supplementary material).

## 4 Discussion

We have proposed a spectral frequency ratio-based coding of neural data to classify cortical state and then investigated whether the simultaneously collected haemodynamic data reflected these cortical state changes. This method has novel aspects for both the analysis of neurovascular coupled data and also for the area of brain state classification. We have validated this method by classifying three experimental datasets—one of concurrent ‘spontaneous’ neural and haemodynamic signals, a second dataset of concurrent neural and haemodynamic signals where 16 s of electrical stimulation was applied to the whisker pad and a final dataset where neural signals were recorded whilst electrical stimulation of the reticular brainstem formation was used to artificially change brain state. We demonstrated that the method of classifying brain state using the ABSC gave important insight into the differences in the both baseline and stimulus evoked haemodynamic changes, especially when compared to unclassified data. The method described also provides an explanation for some of the inherent variability commonly seen in BOLD fMRI signals.

### 4.1 Comparison to previous methods

Neural data was classified according to brain state and the concurrently recorded haemodynamic data was extracted and averaged. During the desynchronised brain state, an increase in the ratio of higher spectral frequencies to lower spectral frequencies was identified. This ratio change corresponded with significant increases in the baseline CBV and blood oxygen saturation, which agrees with the theoretical predictions of Kilner et al. (2004) and the experimental work of Magri et al. (2012). Along with increases in the baseline haemodynamics, decreases in stimulus evoked response magnitudes were again associated with the desynchronised state. The decreases in the evoked response magnitude in desynchronised state are consistent with previous investigations into state evoked haemodynamics (Niessing et al., 2005, Schei et al., 2009, Jones et al., 2008, Berwick et al., 2005), although the corresponding frequency ratio has not previously been documented. This decreased evoked haemodynamic signal could not be identified in the gross unclassified average, and thus the additional haemodynamic information would have been lost, with the resultant average signal reflecting a weaker version of the synchronised average.

This method (ABSC) for classifying brain states, based on relative frequency band vectors, was shown to be both stable and accurate for state classification. Previous brain state classifiers have relied upon clustering neural data, with post-clustering coherence analysis and supervised clustering of data (Gervasoni et al., 2004, Michel et al., 2012). We compared the ABSC to the clustering method put forward by Gervasoni et al. and whilst the accuracy for the data that could be classified was slightly higher than the accuracy of the ABSC, there was much data that the technique was unable to identify, leading to a large overall drop in accuracy. The clustering method was weakest at identifying the desynchronised brain state, which the animals spent less time in. Therefore, with large continuous time periods such as the recordings of 48 h+ that the authors used, we suspect that the performance of the clustering method may improve. The ABSC was also compared to a method of taking an RMS ‘power threshold’ from the raw neural data, and was found to be less accurate than the ABSC or the clustering technique. The poor performance of a RMS ‘power threshold’ technique is unsurprising given the bias that it must entail towards a particular state, unless approximately equal time periods are spent in each of the examined brain states within an experiment.

Whilst these alternative methods can identify distinct brain states, the ABSC represents a different approach. The ABSC has been validated as identifying brain state changes that have robust effects on the concurrently recorded haemodynamics. A further benefit of the ABSC is that it does not rely on human input to detect state, post initialisation. This makes the ABSC an objective classifier. Therefore, a change of state is not required in order to classify subsequent data correctly. Additionally, the ABSC can be modified to search and classify as many states as set by the user. In this paper, we have initialised the classifier to identify only two states: synchronised and desynchronised. Its design however, means that it could adapt to incorporate identifications of multiple states, for example, REM, SWS and awake. A point to note on this matter is that the training dataset would therefore need to include time periods of all the states that were to be classified. Importantly, the ABSC has the potential to classify brain state in real-time. This potential is based upon three qualities that it displays: Firstly, its initialisation parameters are stable over time and across different subjects. Secondly, the ABSC requires a small sample of the dataset to make a classification and finally, the ABSC classification is absolute, as it classifies on an automated point by point basis. The stability of the initialisation parameters was demonstrated by using the single animal training data to classify a much older dataset (Jones et al., 2008) where the state was actively changed by stimulating the brainstem. Further stability of the ABSC was shown by using training datasets from an alternative single subject or a merge of subjects, and yet still achieving high levels of classification accuracy. Therefore, whilst initial parameters may need to be set offline, future experiments could be classified in real-time. The small number of neural sampling points required to give high accuracy classification of experimental trials was demonstrated by the ABSC achieving similar levels of accuracy in single trial brain state prediction when using 10, 5 and 1 s as the time period of data given to the ABSC to classify. With accurate online classification, more understanding of the data in single trial experiments is possible as differences in the data from the brain state rather than the experimental manipulation are more likely to be identified.

### 4.2 Methodological considerations

The initialisation of the ABSC requires careful input. For instance, a compromise of accuracy over efficiency can be made by selecting a larger sliding window, or a larger step size. We also selected the five most frequently occurring model vectors to initialise the ABSC, as this captured a large amount of the calculated vector variance, whilst still making the comparisons with the dataset vectors not too computationally intensive. However, additional vectors could be selected to capture more of the variance if the speed of classification was of less importance. Combining additional vectors with a multicore CPU or graphical processing units (GPU, e.g. with 1280 cores) could potentially optimise both the speed and the accuracy of the ABSC.

We used a urethane anaesthetised rodent model, as urethane is thought to produce similar physiological patterns to the sleep cycle (Clement et al., 2008, Pagliardini et al., 2013), and certainly shows irregular fluctuating periods of synchronised and desynchronised brain states. Urethane is also a stable anaesthetic, causing minimal changes in the ratios of neurotransmitter levels compared to other anaesthetics such as propofol, ketamine or isoflurane (Hara and Harris, 2002) and with minor cardiovascular effects (Maggi and Meli, 1986). Urethane is therefore a favourable choice of anaesthetic for investigating the changes in haemodynamics that occur from spontaneous variations in neural activity.

### 4.3 Implications for future research

An understanding of neurovascular coupling during different brain states may be a vital prerequisite for uncovering the aetiology of neurodegenerative diseases such as dementia (D'Esposito et al., 2003, Iadecola, 2004, Kövari et al., 2007), hypertension (Kazama et al., 2003, Calcinaghi et al., 2013) and ischemic stroke (Shin et al., 2006, Lin et al., 2011), because inferred differences in stimulus evoked haemodynamics or the underlying activity may be due to differences in brain state alone. This manuscript gives a robust method for investigating and understanding how differences in brain state affect both baseline and stimulus evoked haemodynamics and this understanding may therefore benefit research into the aetiology of neurodegenerative diseases.

The ABSC also shows potential as a tool to enhance blood based neuroimaging techniques such as BOLD fMRI, particularly when used with paradigms using single or low numbers of trials. The ability to account for cortical state as a component of the trial-to-trial variability in fMRI could then be used to increase the accuracy of the interpretation of experimental data, such as when a decreased haemodynamic response is a function of state, rather than being generated by stimulus evoked neural changes (Boorman et al., 2015). To do this, we suggest including EEG recordings to identify brain state in BOLD fMRI paradigms. Here, the ABSC can potentially use the EEG recording prior to stimulus presentation to classify brain state from periods of data (1–10 s) short enough not to interfere with experiment length. In addition, when considering fMRI experiments that investigate the BOLD signal without a direct measure of neural activity, we have shown that baseline haemodynamic drifts can provide information regarding changes in cortical state. For example, the baseline haemodynamics could be used as an indication of subjects becoming cortically aroused during an experiment, information which would previously have been disregarded as noise.

### 4.4 Conclusion

We present an automatic state classifier, optimised with a training dataset as a method to examine how changes in cortical state affected coupled haemodynamic signals. The ABSC used only neural data, decomposed into five frequency bands examined in relation to one another to classify the state. A ratio-based signature of these frequencies was found that marked periods of desynchronisation. This was then used to group the haemodynamics. When these desynchronised time periods occurred for more than 30 s, they denoted an increase in the baseline CBV and blood oxygen saturation. This increase in baseline haemodynamics was accompanied by a decrease in stimulus evoked haemodynamics. It is therefore our conclusion that the grouping of haemodynamic data by neural brain state is essential for the full understanding of neurovascular coupled datasets. This approach will allow more stable responses with less variance to be extracted from neuroimaging data, increasing the quality of the data interpretation and at the same time reducing the number of subjects required.

## Figures and Tables

**Fig. 1:**
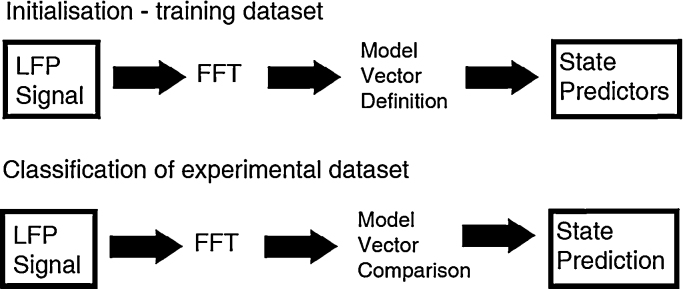
Protocol for classifying brain state from LFP data. The ABSC is initialised on an experiment from a single animal (the training dataset) and then classification of the remaining experiments and animals takes place.

**Table 1:** Showing the comparison between the ABSC and two alternative methods for state classification. Computer spec.: running a 4.2 GHz quad core processor, 16 GB of RAM, with neural data downsampled to 1.53 kHz.

Method	Accuracy			Time taken (s)	Fully automated?
	% Classified correctly	% of data unable to classify	% Total accuracy		
ABSC	*M* = 90.01	0	*M* = 90.01	626.37	Y (after initialisation)
	SD = 7.72		SD = 7.72		
Clustering (Gervasoni et al., 2004)	*M* = 95.09	M = 28.78	*M* = 66.31	3402.17	N—requires identification of cluster centres
	SD = 9.38	SD = 39.04	SD = 36.93		
Power threshold	*M* = 64.88	0	*M* = 64.88	60.14	Y
	SD = 53.04		SD = 53.04		
