# Supplementary material for: A novel method for classifying cortical state to identify the accompanying changes in cerebral hemodynamics
Source: J Neurosci Methods. 2016 Jul 15;267:21–34. doi: 10.1016/j.jneumeth.2016.04.005 (PMC4896992; doi:10.1016/j.jneumeth.2016.04.005)
Supplement: Supplementary file 1 [file mmc1.docx]

**Supplementary Material**

*S1 Invariant Cortical States*


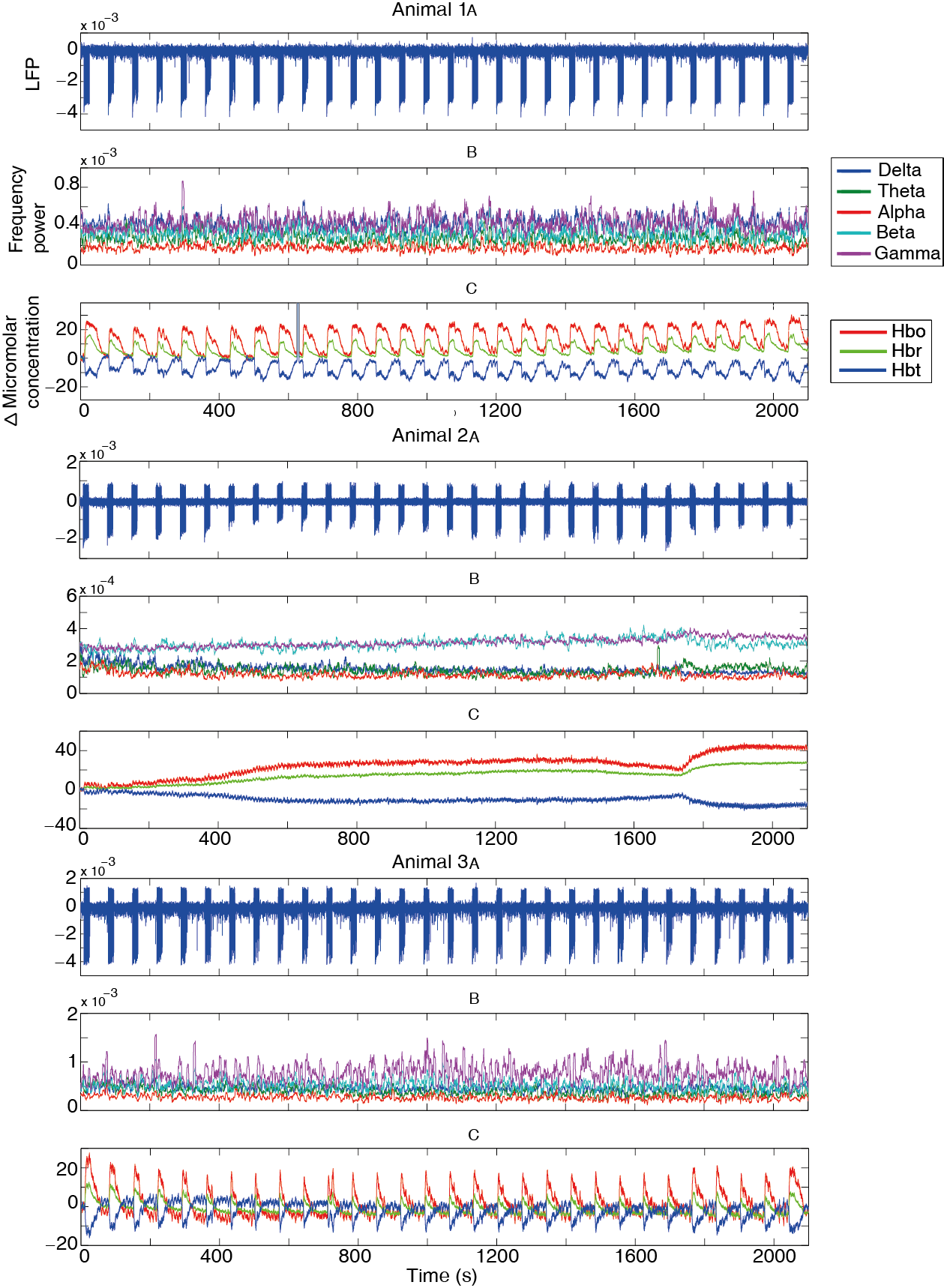


**Fig.A.1: Invariant cortical states.** Three example animals are shown, with the full experiment time series, demonstrating invariant state. Each animal shows (in descending order): neural activity, the frequency power for the classical EEG frequency bands and the concurrent Hbt, Hbo and Hbr for the full time series of 2100s.

The ABSC categorised animals 1 and 3 as being in the synchronised state for the full experiment and animal 2 as being in the desynchronised state. This is in contrast to the ‘expert’ who categorised animals 1 and 3 as desynchronised and 2 as synchronised. The frequency power for the experiments shown on the second line down for animal 2 shows the large amounts of beta and gamma power, relative to alpha, theta and delta. This frequency band pattern is the same as that seen in the desynchronised states in our other experimental data (e.g. in Fig. 4.). The haemodynamic data also supports the classification of the ABSC, for example, for animal 2 the baseline Hbt and Hbo show an increase, whilst the Hbr shows decreases. These observations match with the previous changes in baseline haemodynamics in the desynchronised state reported in Fig. 2. For Animals 1 and 3, we did not see any changes in baseline Hbo, Hbr and Hbt. This observation also matches our previous data in the synchronised state, as seen in Fig.1. It therefore follows that that the ABSC correctly classified the invariant state in the above experiments, whilst the expert could not always do this.

*S2 Animal showing no clear state change after brainstem stimulation*


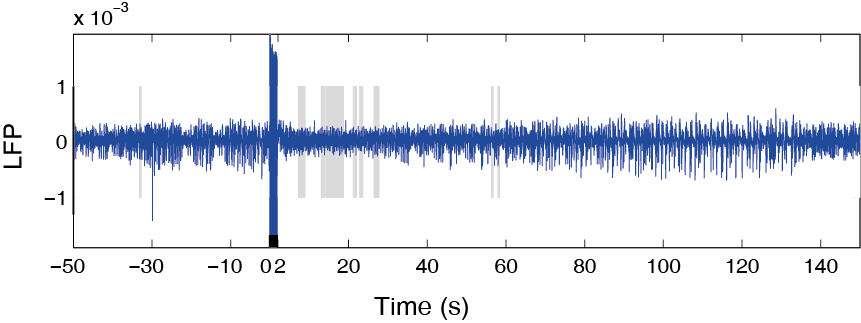


**Fig. A.2:** The application of the ABSC to the fifth animal from the dataset of Jones et al., (2008). Grey sections denote time periods of desynchronisation and white section show sections of synchronisation. The black bar shows the time period of brainstem stimulation and the resulting stimulus artefact can be seen in the LFP.

The ABSC did not find a clear state change after the brainstem stimulation. Whilst some periods of change were found, these were intermittent. The amplitude of the LFPs indicated that over a short period of time, the cortical state returned to synchronised, despite the brainstem stimulation, indicating that the ABSC correctly interpreted the state change as brief and intermittent.
